# Esketamine Regulates Mitophagy through ULK1/FUNDC1 Signaling Pathway to Improve LPS-induced Acute Respiratory Distress Syndrome

**DOI:** 10.2174/0113816128361112250221065359

**Published:** 2025-02-28

**Authors:** Mei Ding, Ping Pei, Weihua Liu, Yingli Cao, Yiqi Weng, Wenli Yu

**Affiliations:** 1 Department of Anesthesiology, Tianjin First Center Hospital, Tianjin, 300192, P.R. China;; 2 Department of Surgery, Nankai University Affiliated Hospital (Tianjin Fourth Hospital), Tianjin, 300222, P.R. China;; 3 School of Medicine, Nankai University, Tianjin, 300071, P.R. China

**Keywords:** Acute respiratory distress syndrome, esketamine, ULK1/FUNDC1 signaling pathway, mitophagy, dihydroethidium, ketamine

## Abstract

**Background:**

As a heterogeneous clinical syndrome, acute respiratory distress syndrome (ARDS) is caused by infection-associated inflammation with limited treatment options. Esketamine possesses anti-inflammatory properties, and it is effective in treating lung diseases.

**Objective:**

This study aimed to unveil the efficacy and mechanism of esketamine in ARDS.

**Methods:**

Lipopolysaccharide (LPS) is widely used to induce inflammatory response in lung injury. The mice model of ARDS in this study was established through the inhalation of LPS. Hematoxylin-eosin (H&E) staining was used to evaluate the pathological changes in the lung tissues of ARDS mice, and the histological index of lung damage was employed. Bicinchoninic acid (BCA) assay kits were utilized to assess the total proteins in bronchoalveolar lavage fluid (BALF), and a hemocytometer was used to count the number of total cells. The pulmonary vascular permeability was detected using Evans blue staining. Western blot was carried out to detect the expressions of tight junction proteins, and enzyme-linked immunosorbent assay (ELISA) detected the release of inflammatory cytokines in BALF and serum. Dihydroethidium (DHE) staining was used to detect reactive oxygen species (ROS) production, and the levels of myeloperoxidase (MPO) and oxidative stress markers were measured using corresponding assay kits. Apoptosis was assessed through terminal deoxynucleotidyl transferase dUTP nick-end labeling (TUNEL) and Western blot. Immunostaining detected the FUN14 domain-containing 1 (FUNDC1) and light chain 3B (LC3B) in lung tissues, and the expressions of autophagy-related proteins were detected using Western blot.

**Results:**

Our data showed that esketamine treatment alleviated LPS-stimulated lung damage, improved pulmonary vascular permeability, and inhibited inflammatory response, oxidative stress, and apoptosis in ARDS mice. Mechanically, esketamine activated mitophagy through UNC-52-like kinase 1 (ULK1)/FUNDC1 signaling pathway. These findings, for the first time, revealed the therapeutic potential of esketamine in treating ARDS.

**Conclusion:**

Collectively, this study revealed the protective role of esketamine against lung injury, inflammation, oxidative stress, and apoptosis in mice with ARDS and revealed the reaction mechanism related to mitophagy.

## 1 INTRODUCTION

Acute respiratory distress syndrome (ARDS), which is a progressive and destructive disease caused by various intra- or extrapulmonary elements, poses great challenges in treatment and rehabilitation [1]. ARDS is characterized by uncontrolled alveolar inflammation, apoptotic lung epithelial/endothelial cells, and pulmonary edema [2, 3]. The present treatment strategies for ARDS patients include mechanical ventilation and fluid management, which have limited treatment efficacy and poor prognosis [4]. Therefore, it is imperative to explore the mechanism of ARDS and develop therapeutic candidates to effectively manage this condition.

**Fig. (1):**
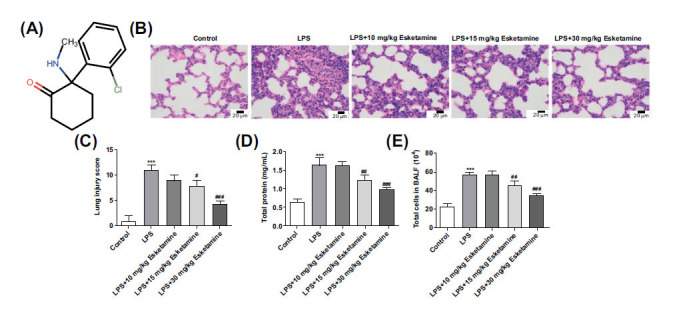
Esketamine treatment improved LPS-induced lung injury in ARDS mice. (**A**) The chemical structure of esketamine. (**B**) The pathological examination using HE staining. (**C**) The lung injury score. (**D**) The total protein in BALF. (**E**) The total cells in BALF. ***p < 0.001 *vs.* Control, ^#^*p* < 0.05, ^##^*p* < 0.01 and ^###^*p* < 0.001 *vs.* LPS. The experiments were replicated three times.

Ketamine, which is a non-competitive N-methyl-d-aspartate receptor antagonist, is often applied as a sedative and non-opioid analgesic outside the ICU because its application has no relation with respiratory depression and can maintain upper airway reflexes [5]. It has been well-documented that ketamine can ameliorate acute lung injury (ALI) induced by high mobility group box-1 protein (HMGB1), which might be realized *via* the toll-like receptor 4 (TLR4) signaling pathway [6]. However, it has been reported that COVID-19 patients with ARDS are more vulnerable to cholestatic liver injury due to long-term infusion of ketamine [7]. Esketamine (Fig. **1A**) is a dextrorotatory isomer of ketamine, and compared with ketamine, esketamine is more controllable and has better efficacy and lower side effects [8]. However, the efficacy and the mechanism of esketamine in ARDS are yet to be clarified.

Excessive production of pro-inflammatory factors is implicated in ARDS advancement [9]. Lipopolysaccharide (LPS), as an endotoxin, can stimulate inflammatory releases, such as interleukin 6 (IL-6), interleukin-1 beta (IL-1β), and tumor necrosis factor-alpha (TNF-α) [10]. LPS stimulation can inhibit UNC-52-like kinase 1 (ULK1) phosphorylation [11]. The phosphorylated activation of ULK1 can activate autophagy in ARDS induced by LPS [12]. Besides, ULK1 also plays a vital role in the activation of mitophagy [13]. FUN14 domain-containing 1 (FUNDC1) is identified to be a novel mitophagy receptor, and the phosphorylation of FUNDC1 by ULK1 promotes the binding of FUNDC1 with light chain 3B (LC3B) to modulate mitophagy [14]. Intriguingly, the Super-PRED database (https://prediction.charite.de/) predicts that ULK1 might be a target of esketamine, and a molecular docking study confirmed the binding of esketamine with ULK1. With this regard, we speculated that esketamine might play a role in LPS-stimulated ARDS *via* ULK1/FUNDC1-mediated mitophagy.

This study aimed to explore the role and reaction mechanism of esketamine in ARDS, which might offer novel therapeutic candidates for treating ARDS.

## 2 MATERIALS AND METHODS

### 2.1 Mice

Forty male C57BL/6J mice (aged 6-8 weeks) were procured from Changzhou Cavens Laboratory Animals Technology Co., Ltd. All mice were kept under standard laboratory conditions with free access to water and standard chow for 1 week for inhabitation. Animal welfare and experimental procedures were conducted strictly according to the National Institutes of Health guide for the care and use of Laboratory animals.

### 2.2 Animal Experiments

The animal experiments were conducted at Zhaofenghua Biotechnology (Nanjing) Co., Ltd. and carried out under a project license (No. IACUC-20240122-01) issued by the ethics board of Zhaofenghua Biotechnology (Nanjing) Co., Ltd. All mice were divided into five groups and each group has 8 mice. For anesthetization, mice were intraperitoneally injected with 50 mg/kg pentobarbital. After that, mice were intratracheally injected with 3 mg/kg LPS (Sigma-Aldrich) and 200 μL of air for the even distribution of LPS within the lungs. Mice in the control group only received an equal volume of normal saline. Esketamine with varying concentrations (10, 15, and 30 mg/kg; Hengrui Pharmaceutical Co., Ltd.; Jiangsu; China) [15] was intraperitoneally injected into mice 1 h after primary LPS or saline administration. Then, 24 h post-treatment, the mice were humanely euthanized. The collected lung tissues were fixed with 4% paraformaldehyde. The surgical interventions and postoperative care for the animal complied with the guidelines and policies established for rodent survival surgery provided by Zhaofenghua Biotechnology (Nanjing) Co., Ltd.

### 2.3 Histopathologic Analysis of Lung Tissues

Following anesthetization, the collected lung tissues were fixed with 4% paraformaldehyde, dehydrated, embedded with paraffin, and processed into 4.5-µm sections. Following, the samples were exposed to hematoxylin and eosin (H&E; Beyotime; Shanghai; China) staining. The degree of lung injury was evaluated according to the following four histological features: alveolar edema, hemorrhage, neutrophil infiltration, and thickness of alveolar walls [16]. Each histological feature was evaluated using scores 0, 1, 2, and 3, respectively, representing normal, mild injury, moderate injury, and severe injury. The total lung injury score was calculated as the sum of these scores.

### 2.4 Collection of Bronchoalveolar Lavage Fluid (BALF)

Following anesthetization, the intratracheal perfusion of 0.8 mL physiological saline and aspiration was performed with an indwelling needle. For the obtaining of supernatants and precipitated cells, the BALF was centrifuged at 2000 rpm at 4°C for 15 min. Bicinchoninic acid (BCA; Thermo Fisher Scientific, Inc.; Shanghai, China) assay kits were utilized to detect the total protein contents in BALF supernatants according to the manufacturer’s instructions. The precipitated cells were re-suspended in phosphate-buffered saline (PBS; 50 μL; Beyotime, Shanghai, China), and a hemocytometer was used to count the number of total cells.

### 2.5 Evaluation of the Lung Edema

The lung was collected from the mouse’s chest, and all extra-pulmonary tissues were removed, followed by the recording of wet weight. Subsequently, the lung tissue was baked at 65°C for 72 h till the weight of the lung remained unchanged. Finally, the dry weight was recorded.

### 2.6 Evans Blue Staining

Thirty minutes before the anesthetization, mice were injected with 200 μL of 5% Evans blue solution (Beyotime, Shanghai, China) *via* the tail vein. Then, the collected lung tissues were weighed and subsequently exposed to the corresponding volume of PBS. The homogenate was prepared through 30 min of centrifugation at 15000 rpm. The collected supernatant was labeled with an equal volume of trichloroacetic acid (Thermo Fisher Scientific, Inc.; Shanghai, China) and incubated overnight, followed by centrifugation at 15000 rpm for 30 min. Finally, the supernatant was collected, and the optical density was read at 610 nm. The amount of Evans blue dye was determined using the standard curve method.

### 2.7 Western Blot

The total proteins were extracted from the lung tissues of mice using radioimmunoprecipitation assay (RIPA) lysis (Beyotime; Shanghai; China) buffer containing protease inhibitors (Beyotime; Shanghai; China). The protein concentration was quantified using BCA assay kits. Separated by sodium dodecyl sulfate-polyacrylamide gel electrophoresis (SDS-PAGE; Bio-Rad Laboratories, Inc.), the proteins were transferred to polyvinylidene fluoride (PVDF; MilliporeSigma) membranes. Inhibited by 5% bovine serum albumin (BSA; Thermo Fisher Scientific, Inc.; Shanghai, China), the membranes were incubated with the primary antibodies specific to occludin, zonula occludens-1 (ZO-1), B cell lymphoma-2 (Bcl-2), Bcl-2 associated X (Bax), cleaved-caspase3, phosphorylated (p)-ULK1, ULK1, COX IV or GAPDH overnight at 4°C. On the next day, the membranes were labeled with horseradish peroxidase (HRP)-conjugated secondary antibodies for 3 h at room temperature. The protein blots were visualized by enhanced chemiluminescence (ECL; EMD Millipore) reagent, and the grey density was analyzed using Image J software.

### 2.8 Cytokine Analysis by Enzyme-linked Immunosorbent Assay (ELISA)

The levels of IL-6, IL-1β, and TNF-α in collected BALF and serum were detected using ELISA assay kits according to the manufacturer’s instructions.

### 2.9 Detection of Myeloperoxidase (MPO)

The collected sample lung tissues were centrifuged at 10000 rpm for 10 min to obtain the supernatants. The level of MPO was assessed using corresponding MPO assay kits according to the manufacturer’s instructions. The optical density was determined at 460 nm.

### 2.10 Detection of Reactive Oxygen Species (ROS)

The ROS production was assessed using dihydroethidium (DHE; Thermo Fisher Scientific, Inc.; Shanghai, China) fluorescent probes. The collected sample lung tissues were incubated with 10 μM DHE solution in the dark and then washed with PBS. A fluorescence microscope was used for the observation of images.

### 2.11 Measurement of Oxidative Stress Markers

The concentrations of malondialdehyde (MDA), glutathione peroxidase (GSH-Px), and superoxide dismutase (SOD) in lung tissues were detected using commercial assay kits procured from Nanjing Jiancheng Bioengineering Institute according to the manufacturer’s instructions. In brief, the homogenate was prepared through the addition of physiological saline, followed by centrifugation at 3000 rpm for 10 min. Finally, the obtained supernatant was used for detection.

### 2.12 Terminal Deoxynucleotidyl Transferase dUTP Nick-end Labeling (TUNEL)

The apoptosis level of lung tissues was assessed using TUNEL assay according to the manufacturer’s instructions. In brief, the collected lung tissues were fixed with 4% paraformaldehyde, dehydrated with ethanol, embedded with paraffin, and then cut into 5 μm-thick sections. Subsequently, the sections were labeled with TUNEL reagent (Beyotime; Shanghai; China) for 30 min according to the manufacturer’s instructions. Finally, the images of TUNEL-positive nuclei were observed using an optical microscope.

### 2.13 Immunostaining and Quantitative Analysis

The prepared lung tissues with a thickness of 10 µm were fixed with cold methanol, permeated with 0.5% Triton X-100, and then blocked by 1% BSA for 2 h. For the immunodetection of FUNDC1 and LC3B, the collected tissue samples were incubated with primary antibodies specific to FUNDC1 and LC3, followed by the incubation with secondary antibodies. 4'-6-Diamidino-2-phenylindole (DAPI; Beyotime; Shanghai; China) was used for nucleus staining, and the images were observed using a microscope. Finally, the immunofluorescence signal was quantified using ImageJ software.

### 2.14 Molecular Docking

The 3D structure of ULK1 (PDB ID:4WNO) was obtained from the PDB database (https://www.rcsb.org/), and the files of esketamine were procured from the PubChem database (https://pubchem.ncbi.nlm.nih.gov/). ULK1 protein structure was prepared *via* removing water and expurgating any irrelevant small ligands. Molecular docking simulation was performed with AutodockTools 4.2 using Lamarckian Genetic Algorithm, and the virtual docking model was visualized *via* Pymol 3.0 software.

### 2.15 Statistical Analysis

The collected data were processed with GraphPad 8.0 software and expressed as mean ± standard deviation. For the exhibition of differences, a one-way analysis of variance (ANOVA) with a post-hoc Tukey's test was carried out. *P*<0.05 indicated statistical significance.

## 3 RESULTS

### 3.1 Esketamine Treatment Improved LPS-induced Lung Injury in ARDS Mice

The chemical structure of esketamine is presented in Fig. (**1A**). ARDS is mainly manifested by diffuse alveolar injury, lung edema formation, and excessive inflammation [17]. H&E staining was used for histopathologic analysis, and it was found that the lung tissues in the LPS group showed severe infiltration of inflammatory cells, hemorrhage, and increased thickness of alveolar septal, which were then concentration-dependently alleviated by esketamine (Fig. **1B**). The lung injury score, total proteins in BALF, and total cells in BALF were also assessed to estimate lung injury. It was noted that esketamine treatment decreased the lung injury scores (Fig. **1C**), total proteins in BALF (Fig. **1D**), and the number of total cells in BALF (Fig. **1E**).

### 3.2 Esketamine Treatment Improved Pulmonary Vascular Permeability in LPS-induced ARDS Mice

**Fig. (2):**
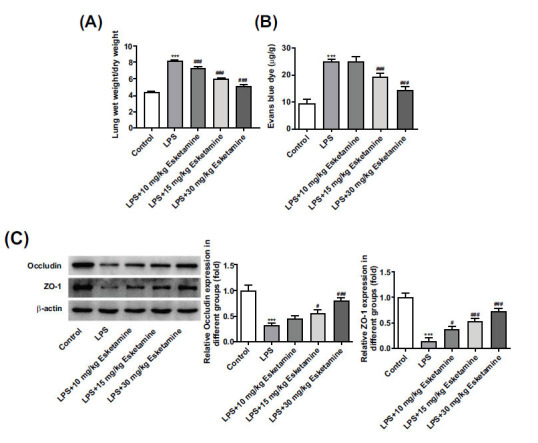
Esketamine treatment improved pulmonary vascular permeability in LPS-induced ARDS mice. (**A**) The pulmonary W/D weight ratio. (**B**) Evans blue staining detected pulmonary vascular permeability. (**C**) The expressions of tight junction proteins were detected using a Western blot. ***p < 0.001 *vs.* control, ^#^*p* < 0.05 and ^###^*p* < 0.001 *vs*. LPS. The experiments were replicated three times.

Increased pulmonary vascular permeability is a major pathological characteristic of ARDS, and it might induce pulmonary edema. The severity of lung edema might be assessed by the lung wet/dry (W/D) ratio of the lung tissues [18]. The increased pulmonary W/D weight ratios due to LPS were partially decreased following the treatment of esketamine (Fig. **2A**). Results of Evans blue staining demonstrated that LPS induction increased the pulmonary vascular permeability in lung tissues, which was subsequently reduced by esketamine (Fig. **2B**). The integrity of tight junctions is important to maintain pulmonary vascular permeability [19]. Tight junctions are composed of a variety of transmembrane proteins, including occludin and ZO-1. Considering this, we performed a Western blot to detect the expressions of these proteins, and it was found that the increased contents of occludin and ZO-1 in lung tissues because of LPS stimulation were concentration-dependently decreased by esketamine treatment (Fig. **2C**).

### 3.3 Esketamine Treatment Alleviated Inflammatory Levels and Oxidative Stress in LPS-induced ARDS Mice

**Fig. (3):**
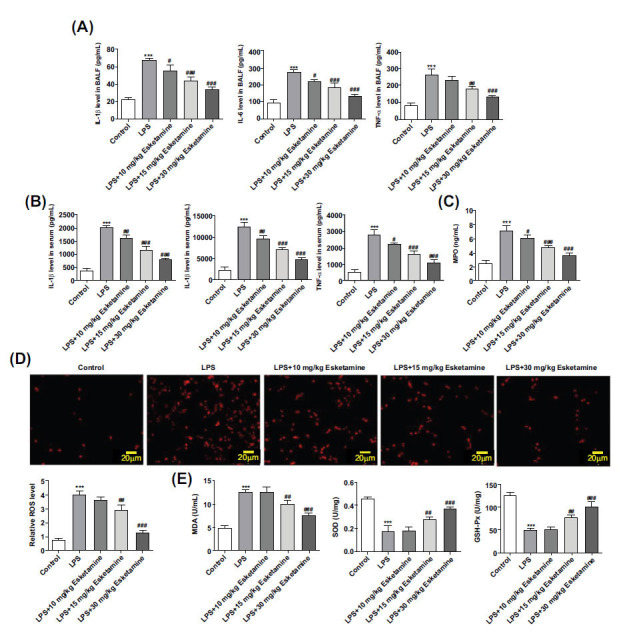
Esketamine treatment alleviated inflammatory levels and oxidative stress in LPS-induced ARDS mice. (**A**, **B**) The levels of inflammatory cytokines were detected using ELISA. (**C**) The MPO level was detected using MPO assay kits. (**D**) The ROS concentration was detected using DHE staining. (**E**) The levels of oxidative stress markers were detected using corresponding assay kits. ***p < 0.001 *vs.* control, ^#^*p* < 0.05, ^##^*p* < 0.01 and ^###^*p* < 0.001 *vs.* LPS. The experiments were replicated three times.

It has been reported that ARDS results from overwhelming pulmonary inflammation [20]. With this regard, we detected the levels of inflammatory cytokines in BALF and serum using ELISA. The results showed that LPS induction significantly increased the levels of IL-6, IL-1β, TNF-α, and MPO, which were subsequently reduced following the treatment of esketamine (Figs. **3A**-**C**). Oxidative stress was found to be related to ARDS pathogenesis [21]. The efficacy of esketamine on oxidative stress in ARDS mice was explored through the measurement of ROS and corresponding oxidative stress markers. The results showed that LPS induction increased ROS and MDA concentration, whereas it decreased GSH-Px and SOD concentration, which were subsequently reversed by esketamine treatment (Figs. **3D**-**E**).

### 3.4 Esketamine Treatment Alleviated Apoptosis in LPS-induced ARDS Mice

**Fig. (4):**
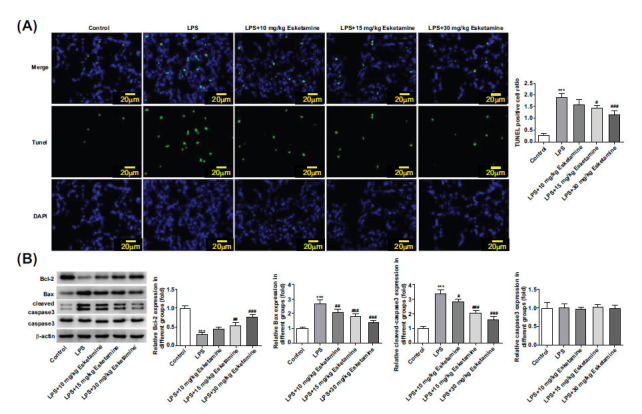
Esketamine treatment alleviated apoptosis in LPS-induced ARDS mice. (**A**) The apoptosis was detected using TUNEL. (**B**) The expressions of apoptosis-related proteins were detected using a Western blot. ***p < 0.001 *vs.* control, ^#^*p* < 0.05, ^##^*p* < 0.01 and ^###^*p* < 0.001 *vs.* LPS. The experiments were replicated three times.

Apoptosis plays an important role in the progression of ARDS [22]. The impact of esketamine on apoptosis was also explored through TUNEL and Western blot. Results of TUNEL revealed that the promoted apoptosis due to LPS induction was dose-dependently suppressed by esketamine (Fig. **4A**). Bcl-2 is an apoptosis inhibitor, and Bax is an apoptosis promoter. We also used western blot to detect the expressions of apoptosis-related proteins, and the results showed that LPS stimulation reduced Bcl-2 expression, whereas it increased Bax and cleaved-caspase3 expressions in LPS-induced ARDS mice. In contrast, esketamine treatment exhibited opposite impacts on these proteins, evidenced by increased Bcl-2 expression and decreased expressions of Bax and cleaved-caspase 3 in the lung tissues of ARDS mice receiving esketamine treatment (Fig. **4B**).

### 3.5 Esketamine Activated Mitophagy *via* ULK1/FUNDC1 Signaling Pathway

**Fig. (5):**
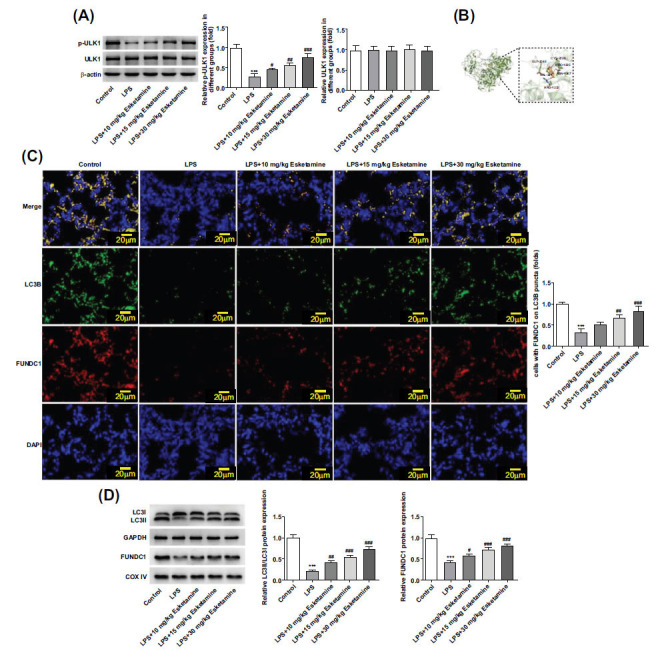
Esketamine activated mitophagy *via* the ULK1/FUNDC1 signaling pathway. (**A**) The expression of ULK1 was detected using a Western blot. (**B**) Molecular docking study. (**C**) The immunofluorescence co-staining for FUNDC1 (green) with LC3B (red) of mice lung tissues. (**D**) The expressions of autophagy-related proteins were detected using a Western blot. ***p < 0.001 *vs.* control, ^#^*p* < 0.05, ^##^*p* < 0.01 and ^###^*p* < 0.001 *vs.* LPS. The experiments were replicated three times.

As reported, ULK1 is implicated in the development of ARDS. The protein expression of ULK1 in lung tissues was assessed using Western blot, and it was found that LPS induction significantly reduced p-ULK1 expression, which was concentration-dependently increased by esketamine (Fig. **5A**). A molecular docking study confirmed that esketamine could target the ULK1 protein (Fig. **5B**). Previous studies have evidenced that mitophagy plays a crucial role in the development of ARDS, and targeting mitophagy is an effective strategy to alleviate ARDS [23]. The activated ULK1 facilitates the interaction of FUNDC1 with LC3 to initiate mitophagy [24]. Considering this, the colocalization experiments were performed, and the results showed that esketamine stabilized the FUNDC1 and LC3B protein complex, indicating that esketamine might initiate mitophagy *via* inducing ULK1/FUNDC1 signals (Fig. **5C**). In addition, Western blot was performed to detect the expressions of autophagy-related proteins, and it was found that LPS induction significantly decreased the expressions of LC3II/LC3I and FUNDC1 in ARDS mice, which were then concentration-dependently increased by esketamine (Fig. **5D**).

## 4 DISCUSSION

ARDS is a primary cause of acute respiratory failure, and it has a close relation with various organ failures [14]. The ARDS pathogenesis is vague, and effective treatment methods are limited, contributing to an unpleasant prognosis for patients with ARDS [25]. Considering this, it is profoundly essential to explore the pathogenesis of ARDS and develop novel therapeutic candidates for ARDS. Our data presented that esketamine could improve LPS-stimulate lung injury and suppress the inflammatory response, oxidative stress, and apoptosis in ARDS mice. Mechanically, esketamine triggered mitophagy *via* ULK1/FUNDC1 signaling pathway. This study preliminarily demonstrated the efficacy and mechanism of esketamine in ARDS, showing the great potential of esketamine as a promising drug candidate for ARDS.

Previous research has shown that LPS induction can cause serious histological changes, including inflammatory cell infiltration, alveolar wall thickness, intra-alveolar exudates, and alveolar congestion [26]. In this study, we also used LPS to treat mice to establish an ARDS model. In LPS-stimulated mice, histological changes could be observed in lung tissue, including the infiltration of inflammatory cells, hemorrhage, and increased thickness of alveolar septal. However, following the treatment of esketamine, these changes were alleviated in a concentration-dependent manner. Apart from that, esketamine treatment also reduced the total protein and cells in BALF and decreased pulmonary W/D weight ratio, suggesting the protective role of esketamine in pulmonary damage induced by LPS.

Increased pulmonary vascular permeability is considered the critical pathophysiological characteristic of ARDS and has been identified to act as a quantitative diagnostic criterion for ARDS [27]. It is well-documented that tight junctions can modulate the permeability of the alveolar epithelium [28]. The tight junctions consist of various transmembrane proteins, such as occludins, and cytoplasmic proteins, such as ZO-1 [29]. Besides, the changes in occludin and ZO-1 expression are closely related to pulmonary tissue permeability [30]. Preclinical research evidenced that tight junctions can be changed by various stimulations, including viral and bacterial pathogens [31, 32]. In this study, it was found that LPS induction increased the pulmonary vascular permeability, accompanied by reduced expressions of occludin and ZO-1 in ARDS mice, and esketamine treatment improved the pulmonary vascular permeability and increased occludin and ZO-1 expressions.

Extensive research has revealed that excessive inflammatory response plays an important role in multiple lung cases and might promote the initiation and advancement of ARDS [33, 34]. The accumulation of ROS is a distinct characteristic of ARDS pathogenesis, which might result in DNA injury, lipid membrane peroxidation, and the activation of pro-inflammatory signals [35]. ROS can also amplify inflammatory lung damage by inducing the release of pro-inflammatory substances [36]. The existing research has shown that ketamine can ameliorate airway inflammation in a mixed-granulocytic murine asthma model [37]. Gonçalves *et al*. reported that ketamine treatment can protect against oxidative damage [38]. Herein, esketamine was found to alleviate LPS-induced inflammation and oxidative stress in ARDS mice, as evidenced by reduced releases of IL-6, IL-1β, TNF-α, and MPO, decreased ROS and MDA concentration, and increased GSH-Px and SOD activities.

Extensive studies have revealed the role of ULK1 and its potential to treat lung diseases [39, 40]. ULK1 expression is downregulated in LPS-induced mouse pulmonary microvascular endothelial cells, and the upregulation of ULK1 can increase LPS-induced autophagy, thereby playing a protective role against LPS-induced ALI [12]. Considering this, we detected ULK1 expression through Western blot analysis and found that LPS induction significantly decreased p-ULK2 expression in ARDS mice. Super-PRED database and molecular docking showed that ULK1 can be targeted by esketamine. In support of this, Western blot analysis further validated that esketamine concentration-dependently increased p-ULK1 protein expression, indicating that esketamine could activate ULK1. Mitochondria participates in various processes, including oxidative stress, apoptosis, and inflammatory response. However, the damage to mitochondria can cause oxidative stress and promote the activation of inflammatory pathways [41]. Mitophagy, a crucial form of selective autophagy, is important for maintaining cellular and mitochondrial homeostasis [42]. In experimental models of ALI/ARDS, mitophagy activation can be observed [43]. In addition, Li and co-workers corroborated that the activation of Parkin-dependent mitophagy can protect against lung injury in ARDS [23]. ULK1 and FUNDC1 are essential for mitophagy, and they modulate mitophagy collaboratively. To be specific, mitophagy receptor FUNDC1 acts as a substrate of ULK1, and ULK1 phosphorylates FUNDC1 to regulate mitophagy [14]. It was found that the activated ULK1 facilitates the interaction of FUNDC1 with LC3 to initiate mitophagy [24]. Considering this, we investigated the protective mechanism of esketamine associated with mitophagy and ULK1/FUNDC1 signaling, and it was revealed that esketamine stabilized the FUNDC1 and LC3B protein complex and increased the expressions of LC3II/LC3I and FUNDC1 in ARDS mice, indicating that esketamine activated mitophagy *via* ULK1/FUNDC1 signaling pathway.

## CONCLUSION

In summary, this study revealed that esketamine could suppress LPS-induced lung damage, pulmonary vascular permeability, inflammation, oxidative stress, and apoptosis in ARDS mice. It was also identified that esketamine activated mitophagy *via* ULK1/FUNDC1 signaling pathway. This study unprecedentedly demonstrated the protective role of esketamine in ARDS and its potential as a promising candidate drug for the treatment of ARDS. However, this study also has limitations. For example, we did not use any mitophagy inhibitors to further validate the detailed role of mitophagy in LPS and esketamine-treated conditions.

## Data Availability

All data included in this study are available by contacting the corresponding author.

## LIST OF ABBREVIATIONS

ARDS: Acute Respiratory Distress Syndrome
BALF: Bronchoalveolar Lavage Fluid
BCA: Bicinchoninic Acid
ELISA: Enzyme-Linked Immunosorbent Assay
H&E: Hematoxylin-Eosin
LPS: Lipopolysaccharide
